# Sialic Acid-Binding Lectin from Bullfrog Eggs Exhibits an Anti-Tumor Effect Against Breast Cancer Cells Including Triple-Negative Phenotype Cells

**DOI:** 10.3390/molecules23102714

**Published:** 2018-10-21

**Authors:** Takeo Tatsuta, Shoko Sato, Toshiyuki Sato, Shigeki Sugawara, Tsuneyoshi Suzuki, Akiyoshi Hara, Masahiro Hosono

**Affiliations:** 1 Division of Cell Recognition Study, Institute of Molecular Biomembrane and Glycobiology, Tohoku Medical and Pharmaceutical University, Sendai, Miyagi 981-8558, Japan; t-takeo@tohoku-mpu.ac.jp (T.T.); ssuga@tohoku-mpu.ac.jp (S.S.); 2 Department of Pharmaceutical Sciences, Tohoku Medical and Pharmaceutical University, Sendai, Miyagi 981-8558, Japan; shoko-s@tohoku-mpu.ac.jp (S.S.); tusuzuki@tohoku-mpu.ac.jp (T.S.); 3 Department of Clinical Pharmacotherapeutics, Tohoku Medical and Pharmaceutical University, Sendai, Miyagi 981-8558, Japan; hachikuma81@gmail.com (T.S.); hara-a@tohoku-mpu.ac.jp (A.H.)

**Keywords:** lectin, sialic acid-binding lectin, ribonuclease, breast cancer, ErbB family

## Abstract

Sialic acid-binding lectin from *Rana catesbeiana* eggs (cSBL) is a multifunctional protein that has lectin and ribonuclease activity. In this study, the anti-tumor activities of cSBL were assessed using a panel of breast cancer cell lines. cSBL suppressed the cell growth of all cancer cell lines tested here at a concentration that is less toxic, or not toxic at all, to normal cells. The growth suppressive effect was attributed to the cancer-selective induction of apoptosis. We assessed the expressions of several key molecules associated with the breast cancer phenotype after cSBL treatment by western blotting. cSBL decreased the expression level of estrogen receptor (ER) α, while it increased the phosphorylation level of p38 mitogen-activated protein kinase (MAPK). cSBL also suppressed the expression of the progesterone receptor (PgR) and human epidermal growth factor receptor type 2 (HER2). Furthermore, it was revealed that cSBL decreases the expression of the epidermal growth factor receptor (EGFR/HER1) in triple-negative breast cancer cells. These results indicate that cSBL induces apoptosis with decreasing ErbB family proteins and may have great potential for breast cancer chemotherapy, particularly in triple-negative phenotype cells.

## 1. Introduction

Lectins are proteins that bind to specific carbohydrate structures. They exist universally in plants, microorganisms, and animals, and have great potential for cancer therapy [1]. Even though only lectins play a role in recognizing sialic acids, i.e., sialic acid-binding lectins (SBLs), there are several lectins that reportedly have anti-tumor effects, such as *Maackia amurensis* seed lectin (MASL) [2], *Polygonatum odoratum* lectin (POL) [3], and *Haliotis discus discus* lectin (HddSBL) [4]. Sialic acids on the plasma membrane are generally observed to be linked to the terminal position of the carbohydrate groups of glycoproteins and glycolipids and have roles in the conformation, recognition, or binding of glycomolecules [5]. Given that altered sialylation is closely associated with malignant phenotypes, including metastasis and invasiveness [6,7], exploration of the effects of SBLs in cancer therapy is a field of great interest for basic studies, and also for clinical researchers.

The 12 kDa protein isolated from *Rana catesbeiana* oocytes was found to be a cell agglutinin [8] of many kinds of cancer cells, but not normal cells. These agglutinations were shown to be inhibited by the sialic acid-containing complex, but not by their asialo-derivatives and, thus, the protein was named *R. catesbeiana* sialic acid-binding lectin (cSBL) [9]. Subsequent analyses revealed that it is homologous to the ribonuclease (RNase) A superfamily and it has substantial RNase activity [8,10,11]. An RNase purified from *R. catesbeiana* oocytes collected in Taiwan by Liao et al., and named RC-RNase, was found to be identical to cSBL [12,13]. Therefore, this interesting SBL is now consequently also called a leczyme (lectin + enzyme) [14,15].

Breast cancer is a molecularly heterogeneous disease [16]. Currently, the classification of breast cancer is based mainly on the expression of the estrogen receptor (ER), progesterone receptor (PgR), and the overexpression or amplification of human epidermal growth factor receptor 2 (HER2/c-ErbB2). In addition, tumors are characterized by grade and proliferative fraction (most commonly assessed by Ki-67). The intrinsic molecular subtypes of breast cancer are known as luminal A-like (strongly ER and PgR positive, HER2 negative, with lower proliferation markers), luminal B-like (variable degrees of ER/PgR expression, with higher proliferative fraction), HER2-enriched (ER and PgR negative, and HER2 positive) and basal-like (ER, PgR, and HER2 negative), and these are routinely used clinically to classify patients for prognostic predictions and to select treatments [17]. The basal-like subtype includes triple-negative breast cancer [18]. Patients diagnosed with triple-negative breast cancer have a poorer prognosis than HER2 and/or hormone receptor positive groups [19]. Recently, the three additional members of the HER/ErbB family of receptor tyrosine kinases (epidermal growth factor receptor (EGFR)/HER1/c-ErbB1, HER3/c-ErbB3 and HER4/c-ErbB4) have been of particular interest because of their ability to interact with HER2 [20]. Members of ErbB family are critically involved in the development and progression of breast cancer. The overexpression of HER1/EGFR is significantly associated with poor prognosis [21,22]. EGFR is well known as a treatment target for colorectal, head and neck, and non-small cell lung cancers, and is also a therapeutic target for breast cancer [23]. 

Since 2011, the efficacy of cSBL on breast cancer cells has been reported; however, the selectivity of cSBL to some cell lines is controversial. Tseng et al. showed that cSBL induces cell death selectively on ER-positive breast cancer cell lines (MCF7 and ZR-75-1), but not on ER-negative breast cancer cell lines (MDA-MB-231 and ZR-75-30) [24]. Their report indicates that ER is an important target of the RNase activity of cSBL. In contrast, our group has demonstrated that cSBL induces cell death in all cell lines tested in the report including MCF7 (ER-, PgR- and HER2-positive), SK-BR-3 (HER2-positive) and MDA-MB-231 (triple-negative) [25]. Here, we tested the effects of cSBL on a larger number of cell lines that represent distinct phenotypes, and also on a normal breast-derived cell line. It was revealed that cSBL exerts its pro-apoptotic effects on all cancer cells, but not on normal breast cells. Furthermore, we found that treatment with cSBL leads to the decrement of HER2 expression, and this reduced expression was also observed with regard to other ErbB family proteins expressed in each cell line. Our results suggest a potential application of cSBL in the treatment of breast cancers, including triple-negative breast cancer.

## 2. Results

### 2.1. Effects of cSBL on Breast Cancer Cell Growth

**Figure 1:**
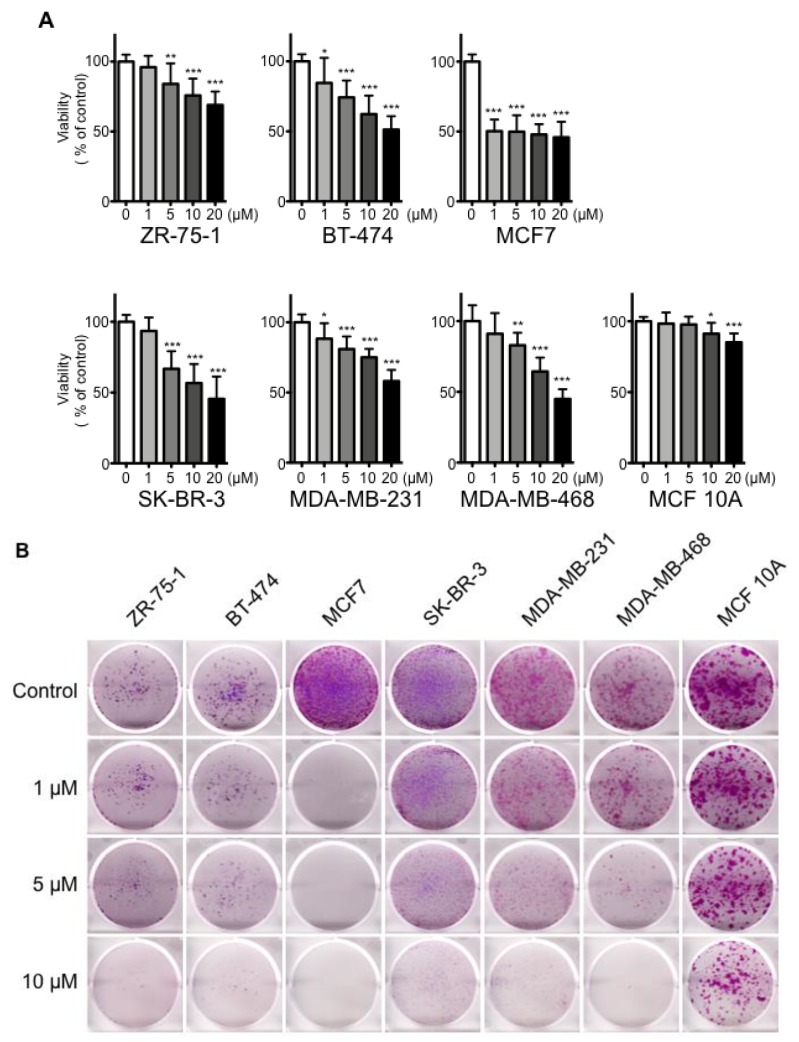

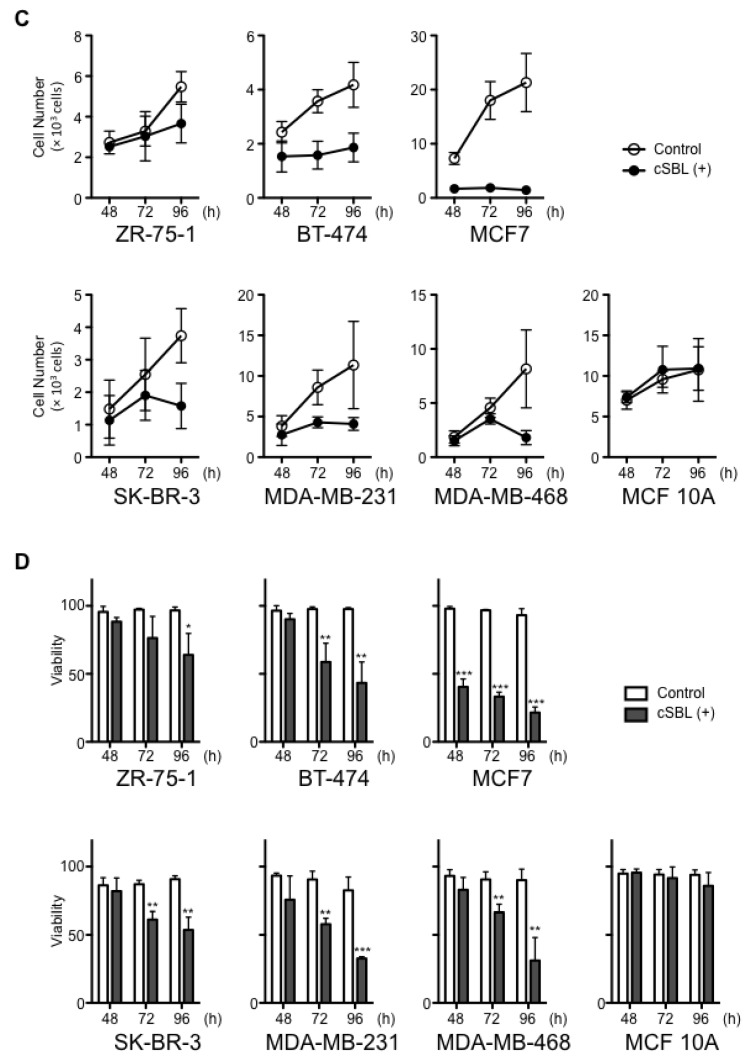
Anti-proliferative effects of *R. catesbeiana* sialic acid-binding lectin (cSBL) against breast cancer cell lines (ZR-75-1, BT-474, MCF7, SK-BR-3, MDA-MB-231, and MDA-MB-468) and a non-tumorigenic epithelial cell line (MCF 10A). (**A**) Viability of cells treated with cSBL. Cells were treated with cSBL (0.2–20 µM) for 72 h. Each data point represents the mean ± SD of three independent WST-8 assays conducted in triplicate. (**B**) The effects of cSBL on the clonogenic potential of the cells. Colony assays were performed on the cells in the absence or presence of cSBL (1, 5, and 10 µM) for 72 h followed by incubation in cSBL-free medium for 7–28 days. Then, the cells were fixed with 2% paraformaldehyde and stained with crystal violet. Representative images from three independent experiments are shown. Cell growth (**C**) and viability were determined by trypan blue assay (**D**) of the cells treated with or without cSBL. Cells were treated with cSBL (10 µM) for 48–96 h. Both live and dead cell numbers were counted by TC10. Each data point represents the mean ± SD of three independent assays. For both graphs, * *p* < 0.05. ** *p* < 0.01. *** *p* < 0.001.

To evaluate the impact of cSBL on breast cancer cell growth, we first examined the effects of cSBL on cell proliferation in several breast cancer cell lines and a normal breast cell line by WST assay. The immortalized human mammary epithelial cell line, MCF 10A, was used as the normal breast cell line due to its non-tumorigenic origin [26]. The characteristic features of each cell lines used in this study are summarized in Table 1. As shown in Figure 1A, treatment with cSBL resulted in a significant reduction in the proliferation of breast cancer cells. In contrast, cSBL hardly showed any inhibitory effect on the proliferation of normal breast cells. Although statistical differences were detected at 10 and 20 µM treatments with cSBL in MCF 10A cells, the viabilities were kept as 91% and 85%, respectively. The viabilities of each cell line with treatment of 20 µM cSBL were as follows: ZR-75-1, 69%; BT-474, 51%; MCF7, 45%; SK-BR-3, 46%; MDA-MB-231, 52%; MDA-MB468, 40%; and MCF 10A, 85%. MCF7 cells were shown to be the most sensitive to cSBL and the inhibitory effect was observed from relatively low concentrations, such as 1 µM (viability: 50%).

Next, we assessed the effects of cSBL on a clonogenic assay. At first, 5 × 10^3^ cells were seeded, and then, the cells were treated with cSBL. cSBL markedly impaired the colony formation of breast cancer cells, whereas it had no profound effect on the growth of normal breast cells (Figure 1B). Untreated control cells of MCF7, MDA-MB-231, and MCF 10A exhibited higher colony forming rates, and ZR-75-1 and BT-474 exhibited low colony forming rates. Since the initial seeding concentration of the cells affected the colony formation efficiency, we also performed the clonogenic assay with different cell seeding concentrations (Supplementary Figure S1). MCF7, SK-BR-3, MDA-MB-231, MDA-MB468, and MCF 10A cells were seeded with 2.5 × 10^3^ cells per well (Figure S1A), ZR-75-1 and BT-474 cells were seeded with 1 × 10^4^ cells per well (Figure S1B). As shown in Figure 1B and Figure S1A and B, similar tendencies were observed in all conditions. Namely, MCF7 showed the highest sensitivity; moderate effects were found in MDA-MB-231 and MDA-MB-468; SK-BR-3, ZR-75-1 and BT-474 exhibited relatively lower levels of sensitivity, but their effects on MCF 10A were limited. Furthermore, lower concentrations of cSBL were assessed with MCF7 and the significant inhibitory effects were confirmed for all cell seeding concentrations tested here, even with treatment of 0.1 µM (Figure S1C,D). 

To obtain further evidence that cSBL only reduced the proliferation of breast cancer cells, we also measured the cell viability with the trypan blue dye exclusion assay (Figure 1C,D), since the assay counts the live and dead cells directly. All untreated control cells continued to proliferate over the experimental period; however, the proliferations of the cancer cells were impaired with cSBL treatment (Figure 1C). cSBL decreased the viability of all cancer cells significantly in a time- dependent manner. In MCF 10A, the effects of cSBL on cell growth and viability were not significant. In summary, cSBL showed concentration- and time-dependent cancer cell growth-suppressing effects. All assays showed a similar tendency, whereby MCF7 exhibited the highest, and MCF 10A exhibited the lowest sensitivity to cSBL. There were no correlations between cSBL effectiveness and the expression statuses of ER, PgR, or HER2. These results suggest that treatment with cSBL selectively leads to a cell growth inhibitory effect on breast cancer cells, regardless of the cancer cell phenotype.

### 2.2. cSBL-Induced Apoptosis

**Figure 2:**
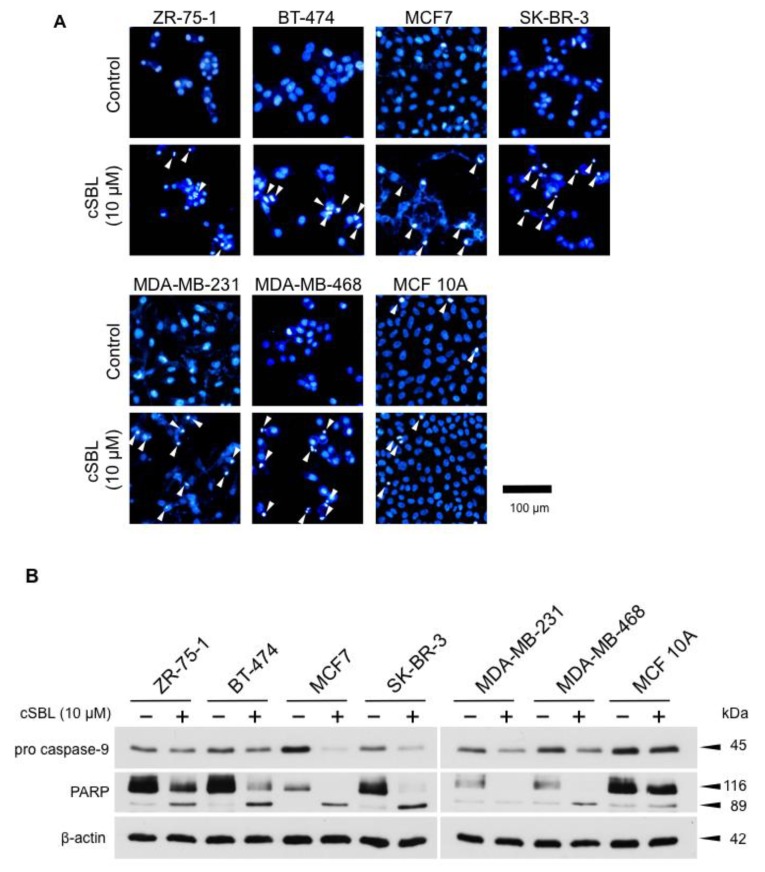
Apoptosis induction in breast cancer cell lines (ZR-75-1, BT-474, MCF7, SK-BR-3, MDA-MB-231, and MDA-MB-468) treated with cSBL. (**A**) Morphological changes of nuclei in cSBL-treated cells. Cells were treated with cSBL (10 µM) for 72 h and stained with Hoechst 33342. Nuclei were observed using a fluorescent microscope. Arrows indicate apoptotic nuclei. Magnification, 20×. (**B**) Cells were treated with cSBL (10 µM) for 72 h, and caspase-9 and PARP cleavage were detected by western blotting.

To investigate further insight into the growth inhibitory effects of cSBL, we assayed apoptotic cell death by detecting morphological and biochemical apoptotic changes. We analyzed nuclear morphological changes by Hoechst staining. As shown in Figure 2A, similar to the effects on cell proliferation, treatment with cSBL led to the induction of chromatin condensation and nuclear collapse in breast cancer cells, whereas it had almost no pro-apoptotic effects on normal cells. Since MCF7 is a type of caspase-3 deficient cell [28], and since the pro-apoptotic effects of cSBL are mainly initiated by caspase-9 activation [29], we analyzed the cleavage of procaspase-9 as an initiative event and that of poly-(ADP-ribose) polymerase (PARP) as an executive event. Treatment with cSBL cleaved the pro-caspase-9 and PARP in breast cancer cells, although the effects were relatively low in ZR-75-1 (Figure 2B). The cleaved form of PARP was detected slightly in MCF 10A treated with cSBL, but the level was comparable with the control. Taken together, these results suggest that cSBL induces apoptotic cell death via caspase cascade activation, particularly in cancer cells. Considering that cSBL decreased viabilities as well as cell numbers of breast cancer cells (Figure 1C,D), it is suggested that cSBL causes the death of cancer cells rather than the arrest of the cell cycle, and the induction of apoptosis is responsible for the cell death.

### 2.3. Effects of cSBL on Cell Survival-Related Molecules

**Figure 3:**
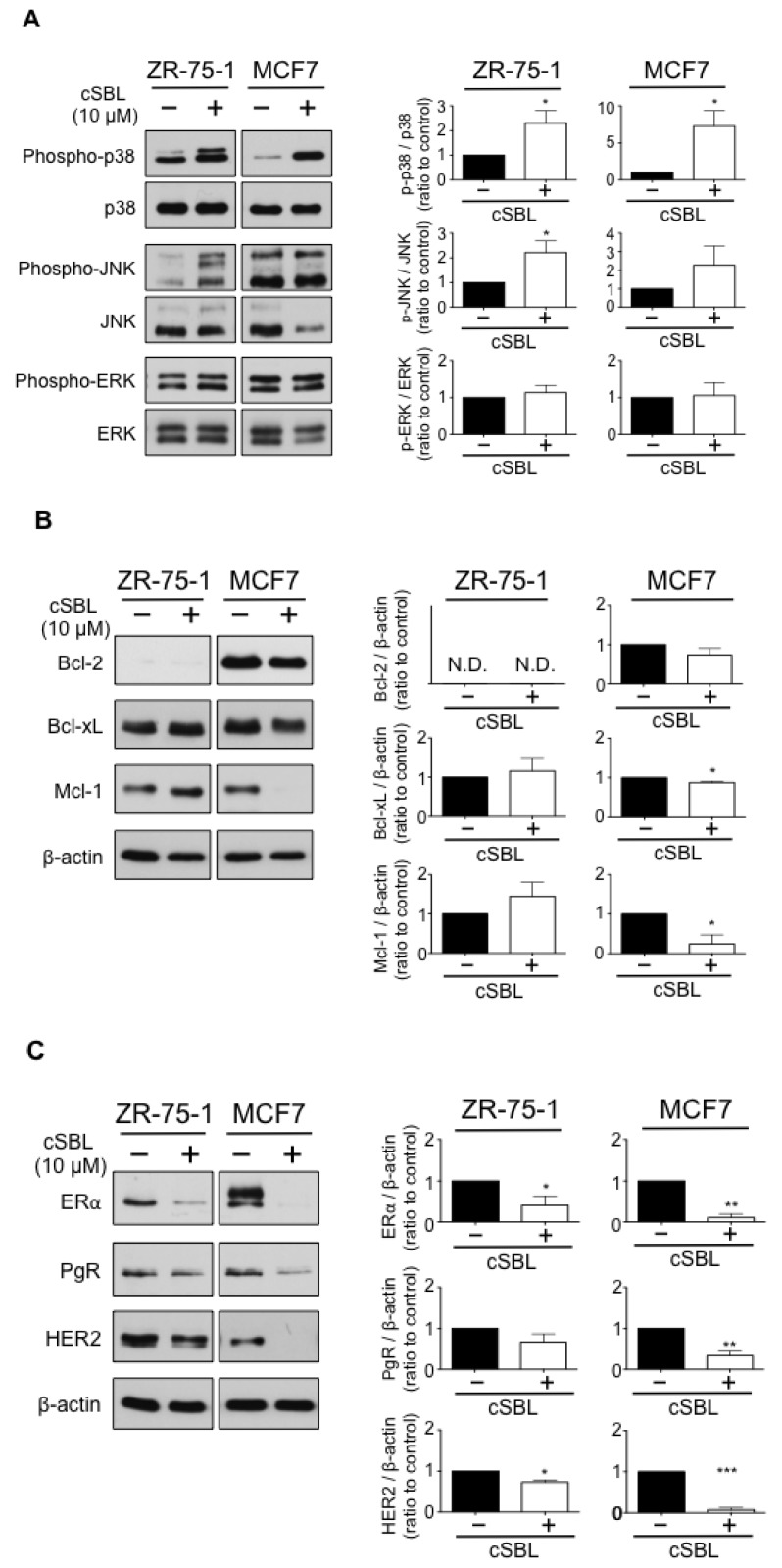
Effects of cSBL on the activations of mitogen-activated protein kinases (MAPKs) (**A**), and expressions of the anti-apoptotic Bcl-2 family (**B**), and breast cancer-related proteins (**C**) in ZR-75-1 and MCF7 cells. Cells were treated with cSBL (10 µM) for 72 h, and the phosphorylation and expression levels of each molecule were detected by western blotting. The values relative to the controls are presented as the mean ± SD of three independent experiments (right graphs). * *p* < 0.05, ** *p* < 0.01, *** *p* < 0.001.

We previously reported that cSBL induces p38 mitogen-activated protein kinase (MAPK) and consequently, activates caspase-3/7 in SBL-induced cell death [25]. Furthermore, Tseng et al., reported that cSBL induces cell death in ER-positive breast tumors through down-regulation of Bcl-2 and ER [24]. To confirm these phenomena and to further investigate cSBL-induced alterations on cell survival-related molecular expressions, we analyzed the phosphorylation of p38, JNK, and ERK MAPKs and protein expressions of the anti-apoptotic Bcl-2 family, as well as ERα, PgR, and HER2 in ER-positive ZR-75-1 and MCF7 cells. As shown in Figure 3A, consistent with previous reports, cSBL induced p38 phosphorylation. A slight phosphorylation of JNK was also observed in ZR-75-1 cells—whereas total JNK expression decreased in MCF7 cells, the phosphorylated JNK level was kept the same as control cells, and relative phosphorylation tended to increase. The phosphorylation status of ERK was not affected by cSBL treatment in both cell lines. Regarding the Bcl-2 family, although statistical significance was not observed, consistent with previous reports, cSBL tended to decrease the expression of Bcl-2 in MCF7, and decrements of Bcl-xL and Mcl-1 were detected in the cells (Figure 3B). In ZR-75-1 cells, Bcl-2 expression was very faint, and BCL-xL and Mcl-1 expression levels were slightly increased. Thus, the effects of cSBL on the anti-apoptotic Bcl-2 family seem to vary depending on the cell line. ERα expression drastically decreased after exposure to cSBL in both cells, as expected (Figure 3C). Interestingly, cSBL also suppressed PgR in MCF7 and HER2 protein expression in both cell lines. These results indicate that cSBL works in an inhibitory manner concerning the protein expressions of key molecules that are implicated in breast cancer phenotypes.

### 2.4. Expressions of ErbB Family Protein in Breast Cancer Cells Treated with cSBL

**Figure 4:**
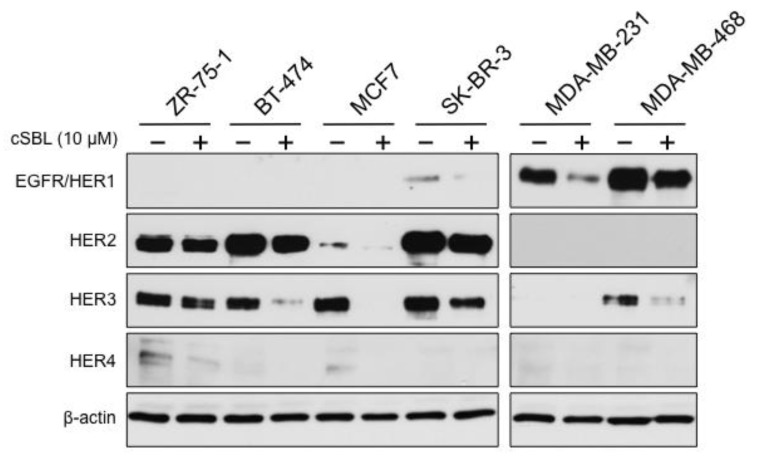
Effects of cSBL on the expression levels of ErbB family proteins in breast cancer cell lines (ZR-75-1, BT-474, MCF7, SK-BR-3, MDA-MB-231, and MDA-MB-468). Cells were treated with cSBL (10 µM) for 72 h and the expression levels of ErbB family in each cell line were detected by western blotting.

Since the ErbB family proteins and their signals were generally attractive therapeutic targets, we evaluated the effects of cSBL on ErbB family expression in all breast cancer cell lines tested here (Figure 4). EGFR/HER1 was predominantly expressed in the triple-negative breast cancer cell lines, MDA-MB-231 and MDA-MB-468, and a faint band was also detected in SK-BR-3. HER2 expression was clearly observed in ZR-75-1, BT-474, SK-BR-3, and slightly in MCF7. HER3 expression was detected in MDA-MB-468 in addition to the four cell lines mentioned above. Slight expression of HER4 was found only in ZR-75-1 and MCF7. Then, the effects of cSBL on the levels of protein expression of these proteins were assessed in each cell line. Surprisingly, treatment with cSBL resulted in decreased protein levels of all ErbB proteins in each breast cancer cell line.

### 2.5. Expressions of EGFR and Its Signaling in Triple-Negative Breast Cancer Cells Treated with cSBL

**Figure 5:**
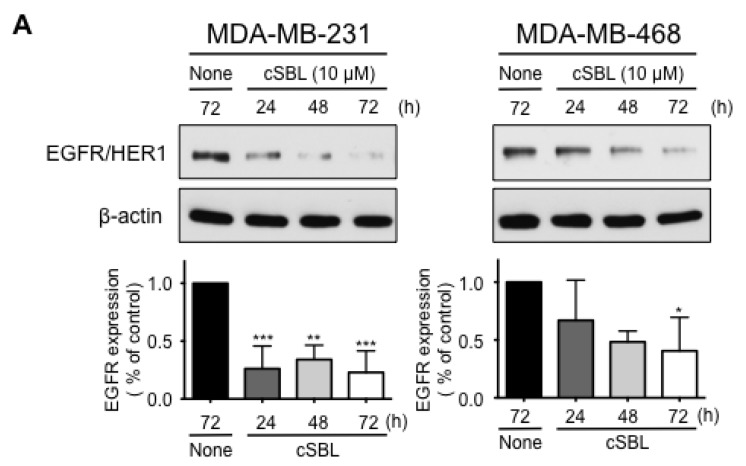

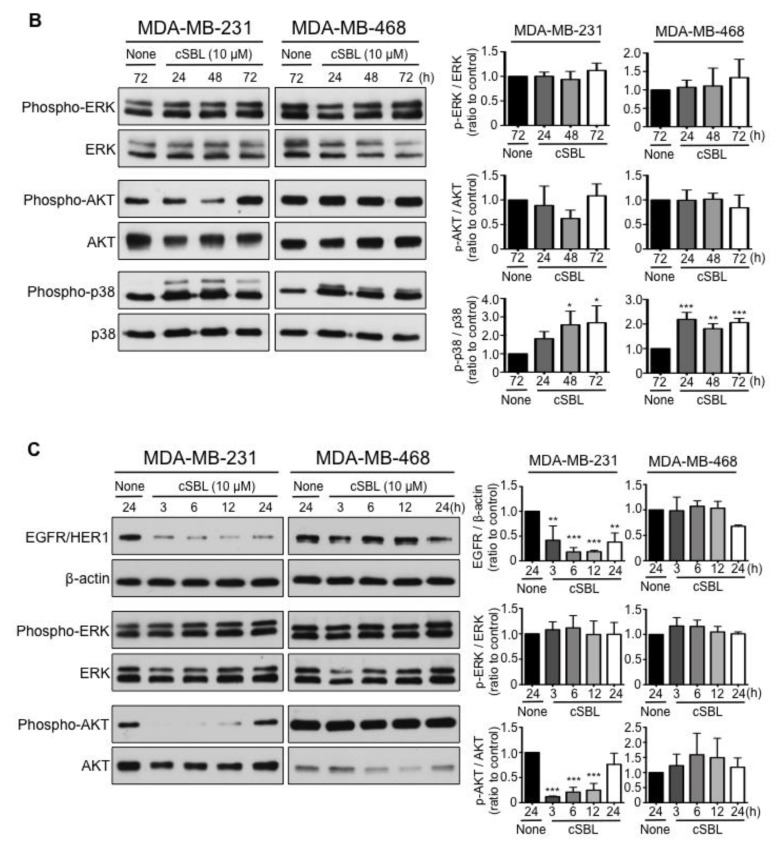
Effects of cSBL on EGFR/HER1 expression and downstream signaling in MDA-MB-231 and MDA-MB-468 cells. (**A**) Cells were treated with cSBL (10 µM) for 24 to 72 h, and the expression levels of EGFR/HER1 in each cell line were detected by western blotting. Densitometric quantification of three independent experiments (mean ± SD) was performed using Image J. (**B**) Cells were treated with cSBL (10 µM) for 24 to 72 h, and the phosphorylation levels of AKT, ERK, and p38 were detected by western blotting. (**C**) Cells were treated with cSBL (10 µM) for 3–24 h and the expression levels of EGFR/HER1 and the phosphorylation levels of AKT and ERK were detected by western blotting. The values relative to the controls are presented as the mean ± SD from three independent experiments (right graphs). For both graphs, * *p* < 0.05; ** *p* < 0.01; *** *p* < 0.001.

Even though some drugs tailored to the breast cancer cell types, such as hormonal agents for ER-positive cells and HER2 targeted agents for HER2 positive cells, have achieved clinical effects to some extent, chemotherapeutic options for triple-negative phenotype breast cancers are very limited. Targeting EGFR/HER1, which is expressed in most patients of triple-negative breast cancer [23], or its signaling is a novel and attractive strategy. Since cSBL was shown to decrease EGFR/HER1 protein expression in triple-negative cell lines, we next sought to explore the downregulation mechanism of EGFR/HER1. Statistically significant reductions in EGFR/HER1 were observed after 24-h and 72-h of treatment with cSBL in MDA-MB-231 and MDA-MB-468, respectively (Figure 5A). 

In an effort to determine the contribution of EGFR/HER1 reduction to the cSBL-induced pro-apoptotic effect, we investigated the activation status of the EGFR signaling pathway. No change was observed in ERK activation in either MDA-MB-231 or MDA-MB-468 cells (Figure 5B). In MDA-MB-231, AKT phosphorylation seemed to slightly decrease after 48-h of treatment with cSBL, but increased after 72-h of treatment compared to the control. The phosphorylation level of AKT was unchanged in MDA-MB-468 cells. In contrast, phosphorylation of p38 increased in both cell lines. 

Since the AKT activation status in MDA-MB-231 cells treated with cSBL was uncertain, we then further observed these events at an earlier time period (from 3 to 24 h of treatment). Even though low EGFR/HER1 levels were observed compared to 24-h untreated control throughout the time points, the level of phosphorylation of AKT gradually increased, and the level at 24-h treatment with cSBL was the same level as that of the 24-h untreated control in MDA-MB-231 (Figure 5C). Thus, it is suggested that AKT phosphorylation increased during the early period in our experimental culture period on cSBL-treated MDA-MB-231, although EGFR/HER1 expression was affected by the treatment. On the other hand, EGFR/HER1 expression was slightly decreased by 24-h of cSBL treatment. The AKT phosphorylation status tended to increase from 3 to 12-h of treatment even though statistical differences were not detected at all time points. 

Taken together, the effects of cSBL treatment on ERK and AKT activation in MDA-MB-231 and MDA-MB-468 cells were not considered to be significant. Similarly, ERK activation was unchanged in ZR-75-1 and MCF7 cells (Figure 3A). Therefore, the EGFR signaling pathway might not be implicated in cSBL-induced apoptosis. P38 phosphorylation, which has been proven to, at least partially, contribute to caspase activation in cSBL-induced apoptosis [25], was observed, and this also supports this prediction. These results indicate that cSBL induces apoptosis to breast cancer cells, including triple-negative cells, accompanied by a decrease in the levels of ErbB family proteins, whereas EGFR signaling is not affected.

## 3. Discussion

In this study, the effects of cSBL on the breast cancer cell growth and on the expression of molecules that play key roles in breast cancer prognosis were evaluated. cSBL significantly suppressed the cell growth of six cancer cell lines representing a variety of phenotypes in ERα, PgR, and HER2 (Table 1 and Figure 1) through the induction of apoptosis (Figure 2). However, the effects of cSBL on normal breast cells were very limited; the viability of MCF 10A stayed over 85% in all concentration ranges tested, and pro-apoptotic changes were not detected under conditions that cause rigid apoptotic changes to cancer cells (Figure 1 and Figure 2). We measured not only the activation of p38 and downregulation of ERα, consistent with previous reports, but also found varied effects on the expression of anti-apoptotic Bcl-2 family proteins depending on the cell types as well as the downregulation of PgR and HER2 in multiple cancer cell lines (Figure 3). Further investigation revealed that cSBL causes a reduction in all ErbB family proteins that are expressed in the breast cancer cell lines (Figure 4). The analysis of EGFR downstream signaling showed that the inhibition of EGFR signaling did not occur in the cSBL treatments (Figure 5). Accordingly, we propose that cSBL triggers apoptosis induction without affecting EGFR signaling, but the downregulation of ERα, PgR, and ErbB receptors are accompanied with cSBL-induced cell death under the experimental conditions used in the current study.

ER and PgR are nuclear hormone receptors whose activation is controlled by ligand binding, kinase activators, or phosphatase inhibitors [30,31,32,33,34,35]. The degradation of ER and PgR has been shown to be under the control of the ubiquitin proteasome system [35,36,37,38,39], and recent research revealed that the phosphorylation of ERα via p38 activation promotes ERα turnover in breast cancer cells [40]. The activation of p38 by cSBL treatment has been observed in leukemia cells [41], mesothelioma cells [42], and some breast cancer cells [25], as well as the cells tested here. Thus, the reduced expression levels of ERα and PgR in cSBL-treated cells may be associated with p38 activation. In the meantime, interaction and stabilization of these hormonal receptors with/by heat shock proteins (HSPs) has also been reported [36,43,44]. We previously found that reducing HSP70 expression attenuates the apoptosis-inducing effects of cSBL [45]. Moreover, treatment with cSBL causes remarkable changes in the localization of HSP70 and HSC70; cSBL treatment has been shown to evoke increases of these proteins in the cytosol prior to the execution of apoptosis in mouse leukemia P388 cells [45]. This implies that HSP70 and HSC70 have important roles in the effects of cSBL, and, also, there is a possibility that cSBL decreases the expression of ERα and PgR through the regulation of HSPs. The implications of proteasome degradation and stabilization by HSPs are common in ErbB receptor turnover. EGFR/HER1 is known to be internalized after ligand binding and part of it is degraded by lysosomes or by the proteasome [46,47]. HSP70 and HSC70 act as key co-chaperones for HSP90 machinery, at least in part, by aiding in client protein recruitment [48]. The HSP70 and HSP90 chaperones work together to target certain client proteins, including EGFR/HER1 for degradation by the ubiquitin-proteasome system, and inhibitory agents targeting HSPs are one of the potential approaches for cancer therapeutics today [49,50]. Further works should be performed to determine the mechanisms by which p38 activation and/or HSPs are involved in the reduction of ERα, PgR, and ErbB receptors in cSBL-treated cells.

It has been reported that cSBL acts as an anti-cancer agent against various human cancer cells including carcinoma (cervical, oral, hepatocellular, and breast), leukemia, lymphoma, mesothelioma, and glioblastoma [24,41,42,51,52,53,54,55,56,57]. Interestingly, although cSBL induces apoptosis in those cancer cells, normal tissue-derived cells, such as fibroblasts, melanocytes, keratinocytes, and mesothelial cells, are relatively insensitive to this agent [53,56,58,59,60]. Moreover, our recent study using human malignant mesothelioma xenograft model mice showed that cSBL exerts significant anti-tumor effects without adverse effects in vivo [61]. It is also noteworthy that the members of the pancreatic-type RNase super-family, including cSBL, show high thermal stability and strong resistance to protein denaturants and to proteases [62,63]. Moreover, they are expected to have lower immunogenicity due to their compact structures and homology to human pancreatic RNases [64]. However, only limited information has been obtained regarding its cancer-selectivity so far. The known factors that affect cell susceptibility are summarized in our previous review [65]. To date, differences in the cSBL binding to the cell surface and its internalization into cytosol were considered to be the main reasons for the selectivity. In the present study, pro-apoptotic effects were observed irrespective of breast cancer cell type, and this is not consistent with previous reports; cSBL is effective towards breast cancer cells, including triple-negative breast cancers. It can be speculated that one of the reasons for this contradiction may be the variance of cell culture conditions. The elements that influence the cell dependency on particular hormones or growth factors, such as FBS, composed of complete medium may be an explanation. Our results also revealed obvious differences in sensitivity between MCF7 and MCF 10A to cSBL. Comparative studies on cSBL binding and/or internalization to these cells would give us evidence for the cancer selectivity of cSBL that could promote the development of novel anti-cancer agents. Furthermore, growth signals, such as the AKT and ERK activation of the cell lines investigated here, seem to be independent of EGFR/HER1 expression. Although involvement of alternative pathways such as PLCγ or STATs activated by EGFR/HER1 in the effects of cSBL needs to be elucidated, our results suggest that applications of cSBL against the cancer cells that depend on the ErbB receptors for growth may provide great benefit as a type of chemotherapy. Moreover, recent studies on triple-negative breast cancer therapeutics have been shown to affect the crucial parts of ErbB receptors [48,66,67]. Tao et al. reported that single treatment with an AKT inhibitor (GDC-0068) or a PI3K inhibitor (GDC-0941), inhibitors for EGFR downstream signaling, activates EGFR/HER1 and HER3 [68]. The blockade of EGFR and HER3 combined with drugs results in superior anti-tumor activity in vitro and in vivo, suggesting the importance of inhibition on both the upstream activation of key molecules and the downstream signaling pathway, due to the cellular response of activating an alternative pathway for cancer cell survival. Thus, the combination with cSBL that reduces the expressions of all ErbB family proteins with drugs such as AKT inhibitors could have a great potential for therapy in breast cancers, including triple-negative phenotype cells.

## 4. Materials and Methods

### 4.1. Reagents

cSBL was isolated using sequential chromatography with Sephadex G75, DEAE-cellulose, hydroxyapatite, and SP-Sepharose, as previously described [9]. The anti-caspase-9 antibody was purchased from Medical and Biological Laboratories Co., Ltd. (MBL; Nagoya, Japan). The anti-PARP (46D11), phospho-p38 (Thr180/182) (D3F9), p38 (D13E1), Phospho-ERK (Thr202/Tyr204) (F13.14.4E), ERK (137F5), Bcl-2 (D55G8), Bcl-xL (54H6), Mcl-1 (D35A5), EGFR/HER1 (D38B1), HER2 (29D8), HER3 (D22C5), HER4/ErbB4 (111B2), phospho-AKT (Ser473) (D9E), AKT (11E7), and phospho-AKT (Ser473), horseradish peroxidase (HRP)-conjugated anti-mouse IgG and anti-rabbit IgG antibodies were purchased from Cell Signaling Technology (Beverly, MA, USA). The anti-β-actin antibody was purchased from Sigma-Aldrich (Merck KGaA, Darmstadt, Germany). The anti-phospho-JNK (pT183/pY185), and JNK antibodies were purchased from BD Biosciences (Franklin Lakes, NJ, USA). The anti-ERα (E115) and PgR (ERP5489) antibodies were purchased from Abcam Biotechnology (Cambridge, UK). 

### 4.2. Cell Culture

The breast cancer cell lines (ZR-75-1, BT-474, MCF7, SK-BR-3, MDA-MB-231, and MDA-MB-468), and a non-tumorigenic epithelial cell line, MCF 10A, were purchased from American Type Culture Collection (ATCC; Manassas, VA, USA). The cancer cells were cultured in Dulbecco’s modified Eagle’s medium (WAKO Pure Chemical Industries, Ltd., Osaka, Japan) supplemented with 10% fetal bovine serum (Biosera, Nuaille, France), 100 U/mL penicillin, and 100 μg/mL streptomycin (Life Technologies, Carlsbad, CA, USA). MCF 10A cells were cultured in mammary epithelial cell growth medium (Lonza, Basel, Switzerland) supplemented with bovine pituitary extract, hydrocortisone, hEGF, insulin (Bullet Kit, Lonza) and 100 ng/mL cholera toxin (WAKO Pure Chemical Industries, Ltd.). All cell lines were cultured in a humidified atmosphere in 5% CO_2_ at 37 °C.

### 4.3. WST Assays 

WST-8 assay was performed to determine the cell viability. Cells (5 × 10^4^ cells/mL) cultured in 96-well plates (100 µL/well) were treated with cSBL (1, 5, 10, and 20 µM) for 72 h. Then, the cells were incubated with Cell Count Reagent SF (Nacalai Tesque Inc., Kyoto, Japan) for 1–4 h. The absorbance of the resulting product at 450 nm was measured, and the background absorbance at 650 nm was subtracted. Experiments were conducted in triplicate.

### 4.4. Clonogenic Assay

The cells were seeded in six-well plates (5 × 10^3^ cells/well) and allowed to adhere overnight. The cells were treated with 1–10 μM cSBL for 72 h. The culture medium was subsequently replaced with cSBL-free culture medium and the cells were incubated for 7–28 more days (7 days: MCF7, MDA-MB-231, MCF 10A; 14 days: SK-BR-3, MDA-MB-468; 28 days: ZR-74-1, BT-474). The colonies were fixed with 2% paraformaldehyde and stained with crystal violet. 

### 4.5. Trypan Blue Dye Exclusion Assay 

A trypan blue dye exclusion assay was also performed to assess the effects of cSBL on cell viability. Cells (5 × 10^4^ cells/mL) cultured in a 24-well plate (500 μL/well) were treated with cSBL (10 μM) for 48, 72, and 96 h. Then, the cells were harvested, and the numbers of live or dead cells were counted by TC10 (Bio-Rad Laboratories, Inc. Hercules, CA, USA) in accordance with to the manufacturer’s instructions. 

### 4.6. Observation of Nuclear Morphology

Cells (5 × 10^4^ cells/mL) cultured in a Cell Carrier-96 Ultra Microplate (100 μL/well) were treated with cSBL (10 μM) for 72 h. Then, cells were stained with 2 μg/mL Hoechst 33342 (Dojindo Laboratories, Kumamoto, Japan) for 1 h. The resulting images were acquired with the high-content analysis system Operetta CLS™ with NA 20× objectives.

### 4.7. Western Blotting

The cells (5 × 10^4^ cells/mL) were cultured in six-well plates (4 mL/well) and were treated with cSBL (10 μM) for the indicated time. Whole cell lysates were prepared using extraction buffer (150 mM NaCl, 10 mM Tris-HCl (pH 7.4), 5 mM EDTA, 1% Nonidet P-40, 0.1% sodium deoxycholate, and 0.1% sodium dodecyl sulfate) supplemented with cOmplete™ Mini EDTA-free protease inhibitor cocktail tablets and PhosSTOP phosphatase inhibitor tablets (each 1 tablet/10 mL; Roche Applied Science, Indianapolis, IN, USA). Soluble proteins were collected, and the protein concentration was measured using a BCA protein assay kit (Thermo Fisher Scientific, Inc., Waltham, MA, USA) according to the manufacturer’s instructions. The proteins were separated using 10% or 14% SDS-PAGE and transferred onto Immobilon-P transfer membranes (EMD Millipore, Billerica, MA, USA). The membranes were sequentially incubated with primary and secondary antibodies diluted in Can Get Signal solution (Toyobo Co., Ltd., Osaka, Japan). The protein bands were detected using ECL Prime Western Blotting Detection Reagent (GE Healthcare, Little Chalfont, Buckinghamshire, UK). The relative density of the protein bands was measured by ImageJ 1.51s software (National Institutes of Health, Bethesda, MD, USA). The experiments were repeated three times.

### 4.8. Statistical Analysis

Results are expressed as means ±SE or SD and are the representative of at least three independent experiments. Statistical comparisons of two groups were made using Student’s *t*-tests and among groups using one-way ANOVA, followed by Bonferroni’s post hoc tests. All calculations were performed using GraphPad Prism version 5.0 (GraphPad Software Inc., San Diego, CA, USA). The significance threshold was *p* < 0.05.

## 5. Conclusions

In summary, our founding suggestion is that cSBL exhibits an apoptosis-inducing effect regardless of the breast cancer cell type and has high cancer selectivity. These effects are accompanied by a decrease in the ErbB family receptors. To explore the effects of cSBL on cancer cells showing ErbB family-dependent growths, a combinatorial study with EGFR signal-targeted drugs, such as AKT inhibitors, should be performed.

## Figures and Tables

**Table 1:** Classification of breast cancer cell lines [16,27].

Cell Line	ER ^1^	PgR ^1^	HER2 ^2^	Subtype
ZR-75-1	+	+	2+	Luminal A
BT-474	+	+	3+	Luminal B
MCF7	+	+	0-1+	Luminal A
SK-BR-3	-	-	3+	HER2
MDA-MB-231	-	-	0-1+	Basal
MDA-MB-468	-	-	-	Basal
MCF 10A	-	-	0-1+	Basal

^1^ Data from mRNA, protein levels [16], and/or immunohistochemical (IHC) analysis [26]; ^2^ Data from IHC analysis [26]. The Allred score was used for HER2.

## Supplementary Materials

The following are available online, Figure S1. Effects of cSBL on clonogenic potential of the cells seeded in distinct cell densities. Colony assays on the cells were performed in the absence or presence of cSBL for 72 h followed by incubation in cSBL-free medium for 7–28 days. The cells were fixed with 2% paraformaldehyde and stained with crystal violet. (A) 2.5 × 10^3^ cells were seeded and allowed to adhere overnight, and then the cells were treated with 1–10 µM cSBL. (B) 1 × 10^4^ cells were seeded and allowed to adhere overnight, and then the cells were treated with 1–10 µM cSBL. 5 × 10^3^ (C) or 2.5 × 10^3^ (D) cells were seeded and allowed to adhere overnight, and then the cells were treated with 0.1 and 0.5 µM cSBL. Representative images are shown.
