# Supplementary figures and images for: Sialic Acid-Binding Lectin from Bullfrog Eggs Exhibits an Anti-Tumor Effect Against Breast Cancer Cells Including Triple-Negative Phenotype Cells
Source: Molecules. 2018 Oct 21;23(10):2714. doi: 10.3390/molecules23102714 (PMC6222625; doi:10.3390/molecules23102714)

**A**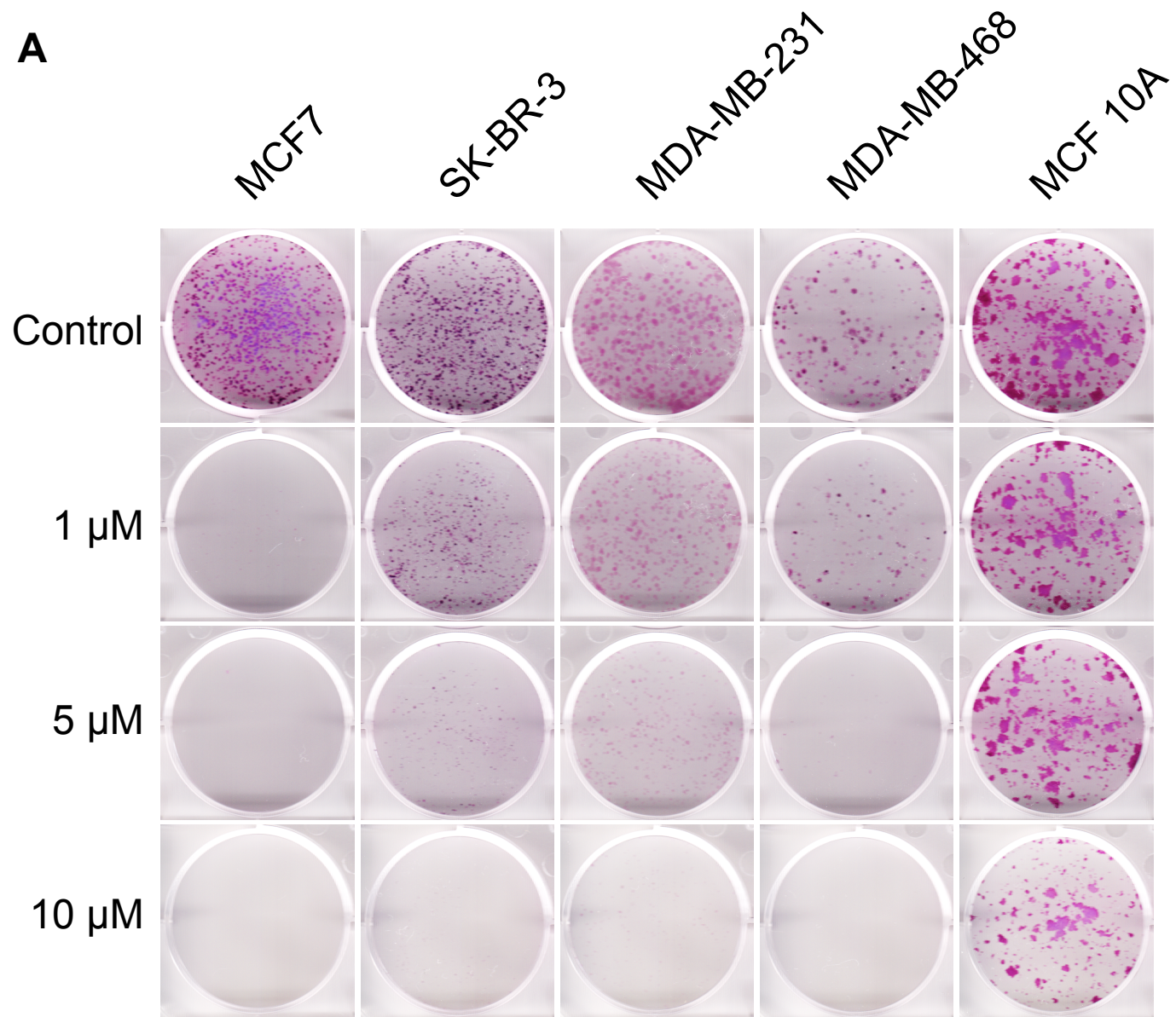

**B**

ZR-75-1  
BT-474

Control

1  $\mu$ M5  $\mu$ M10  $\mu$ M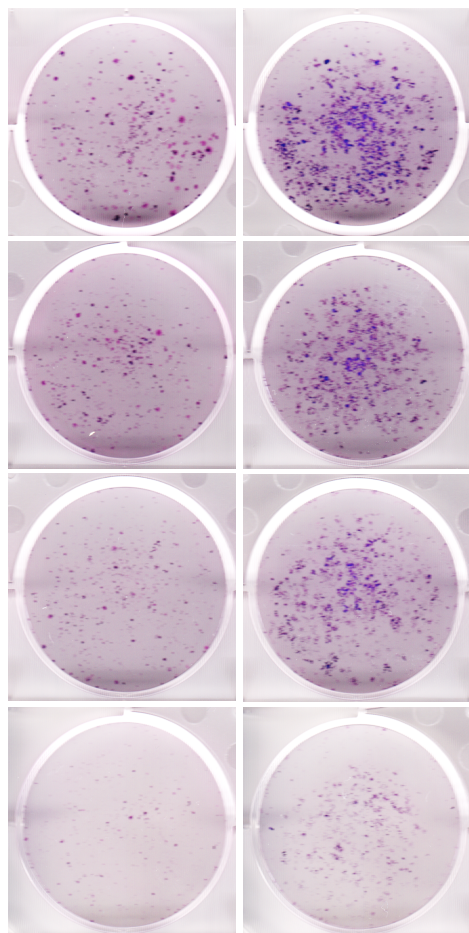**C**

MCF7

Control

0.1  $\mu$ M0.5  $\mu$ M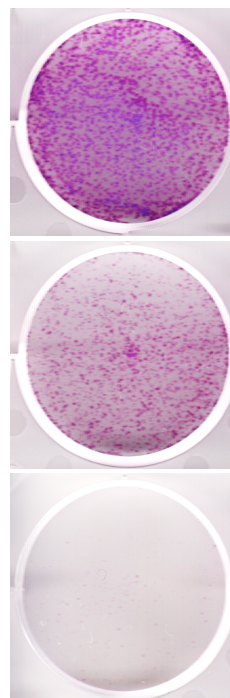**D**

MCF7

Control

0.1  $\mu$ M0.5  $\mu$ M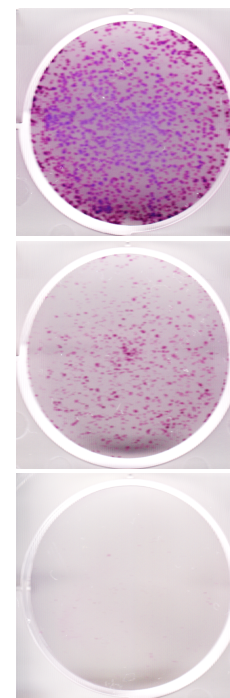

Supplement: Supplementary file 1 [file molecules-23-02714-s001.pdf]
